# Strength Assessment of PET Composite Prosthetic Sockets

**DOI:** 10.3390/ma16134606

**Published:** 2023-06-26

**Authors:** Yogeshvaran R. Nagarajan, Farukh Farukh, Vadim V. Silberschmidt, Karthikeyan Kandan, Radheshyam Rathore, Amit Kumar Singh, Pooja Mukul

**Affiliations:** 1 Institute of Engineering Sciences, School of Engineering and Sustainable Development, De Montfort University, The Gateway, Leicester LE1 9BH, UK; 2 Wolfson School of Mechanical, Electrical and Manufacturing Engineering, Loughborough University, Loughborough LE11 3TU, UK; 3 Department of Mechanical Engineering, Malaviya National Institute of Technology, Jaipur 302017, India; 4 Department of Mechanical Engineering, National Institute of Technology Calicut, Kozhikode 673601, India; 5 Bhagwan Mahaveer Viklang Sahayata Samiti (BMVSS), Jaipur 302017, India

**Keywords:** prosthetic sockets, PET fibre-reinforced composite, woven laminate, knitted composite, tensile testing, ISO socket testing, polypropylene and high-density polyethylene

## Abstract

A prosthesis is loaded by forces and torques exerted by its wearer, the amputee, and should withstand instances of peak loads without failure. Traditionally, strong prosthetic sockets were made using a composite with a variety of reinforcing fibres, such as glass, carbon, and Kevlar. Amputees in less-resourced nations can lack access to composite prosthetic sockets due to their unavailability or prohibitive cost. Therefore, this study investigates the feasibility of polyethylene terephthalate (PET) fibre-reinforced composites as a low-cost sustainable composite for producing functional lower-limb prosthetic sockets. Two types of these composites were manufactured using woven and knitted fabric with a vacuum-assisted resin transfer moulding (VARTM) process. For direct comparison purposes, traditional prosthetic-socket materials were also manufactured from laminated composite (glass-fibre-reinforced (GFRP)), monolithic thermoplastic (polypropylene (PP) and high-density polyethylene (HDPE)) were also manufactured. Dog-bone-shaped specimens were cut from flat laminates and monolithic thermoplastic to evaluate their mechanical properties following ASTM standards. The mechanical properties of PET-woven and PET-knitted composites were found to have demonstrated to be considerably superior to those of traditional socket materials, such as PP and HDPE. All the materials were also tested in the socket form using a bespoke test rig reproducing forefoot loading according to the ISO standard 10328. The static structural test of sockets revealed that all met the target load-bearing capacity of 125 kg. Like GFRP, the PETW and PETK sockets demonstrated higher deformation and stiffness resistance than their monolithic counterparts made from PP and HDPE. As a result, it was concluded that the PET-based composite could replace monolithic socket materials in producing durable and affordable prostheses.

## 1. Introduction

A prosthetic socket is a crucial part of a prosthesis as it connects the residual limb and the prosthetic device. Therefore, it must be designed and fabricated to ensure the appropriate fit and comfort. At the same time, a lower-limb prosthetic socket (PS) undergoes various types of loading due to the weight and gait of the amputee. Hence, understanding the performance of a prosthetic socket is vital to ensure sufficient strength and durability. The World Health Organization estimates that approximately 35–40 million people require assistive devices and services for prosthetic and orthotic applications [1]. Despite technological advancements, many amputees do not have access to prosthetic and orthotic devices, especially in less-resourced nations (LRNs). The primary cause of abandonment of the prosthetic device is the repair and replacement costs. To overcome this, the LRNs rehabilitation providers provide easily accessible material at a shorter distance to reach the clinical centre [2]. Stiffness and strength, durability, manufacturability, and cost are crucial factors in choosing the appropriate material for the fabrication of functional PSs. In LRNs, thermoplastic polymers, such as high-density polyethylene (HDPE) and polypropylene (PP), are the primary candidates for a PS when considering cost and ease of manufacturing. In general, thermoplastic sockets are less durable due to inadequate levels of strength and stiffness to withstand the maximum axial and shear stresses induced in a socket by the amputee’s weight and gait, thus directly affecting comfort and fitting. Therefore, laminated composites of thermosetting resin reinforced with synthetic fibres, such as carbon/glass/Kevlar, were better for manufacturing the PS. Based on the literature analysis, the vacuum-assisted resin transfer technique is the most effective method for producing composite materials suitable for socket production [3]. The vacuum’s involvement in the process guarantees consistent pressure, minimising void formation during consolidation and allowing for the creation of intricate pieces [4,5]. Though these advanced composite materials have excellent mechanical properties, they are prohibitively expensive for amputees in LRNs [6]; a patient-specific socket costs from USD 6000 to USD 20,000 in the initial five years after the amputation [7].

To address the challenges of expensive composites made from synthetic fibres, various studies used natural fibres, such as ramie, pineapple, and banana, to produce bio-based composites [8,9]. Though the fibres are obtained from natural sources, the reinforcements were infused with either epoxy or polyester resin to produce bio-composites that are not bio-degradable, despite using plant-based fibres. Furthermore, many natural-fibre-reinforced composites lack adequate stiffness and strength to contain the limb during prosthetic use. To overcome this issue, hybrid composites were proposed by combining natural fibres (Jute) as well as synthetic fibres (carbon, glass, and perlon) [9]. Regardless of the development of natural composites from conventional sources, the laminates with natural fibres are yet to be deployed in PS for amputees involved in dynamic activities. Numerous studies have been conducted searching for suitable composite materials for socket fabrication. These studies aim to identify the optimal combination of matrix and fibre reinforcement and fibre orientation or producing laminated prosthetic sockets [10,11,12]. For example, Phillips and Craelius examined 24 different laminate combinations for prosthetic applications [10], while Odusote et al. investigated the material properties of pineapple fibre reinforced with polyester and epoxy resin. The ISO 10328 test method is widely practised to assess socket strength since no standard method exists for accessing socket strength [13]. However, only a few studies were conducted to analyse the performance of the manufactured socket concerning the mechanical properties of constituent socket materials.

With advancements in additive manufacturing (AM), a focus was placed on the automated design and development of 3D-printed sockets in the last two decades. Various 3D-printing methods, such as selective laser sintering (SLS) and fused deposition modelling (FDM), were used to manufacture the sockets. However, the work related to the strength evaluation of these 3D-printed sockets was comparatively limited. Recently, some research groups explored the static strength and cyclic loading of 3D-printed sockets for adult and pediatric patients [14,15,16,17]. To produce a patient-specific socket, the geometry of the residuum was obtained via 3D scanning. Then limb geometry was modified using a haptic device or an automated software algorithm, subsequently printing the socket using SLS and FDM process [18,19]. Recent studies demonstrated the promise of producing 3D-printed sockets to meet static strength and cyclic-loading criteria of the ISO standard 10328. However, the material and printing parameters should be tailored for patient-specific 3D printed sockets to achieve a good fit and comfort criteria and also require static structural testing to meet the strength criteria of the ISO standards 10328. AM has a great potential to produce prosthetic sockets with great accuracy and consistency compared to traditionally manufactured ones. Although additive manufacturing has numerous benefits, the strength of 3D printed materials remains a significant limitation to its clinical adoption [20]. To address this concern, researchers have explored various approaches, such as chopped fibre, polymer coating, fill compositing, and particulate additives, to enhance the material’s strength [21,22,23,24]. For example, Isaac A. Cabrera investigated the material behaviour of vacuum-infused fill compositing for AM prosthetic sockets [25]. However, the cost of 3D-printed sockets makes them unsuitable for mass production.

Despite the technological advancements in synthetic and natural fibre-reinforced composites and 3D printing technologies, there is a considerable demand for prostheses worldwide. This demand is further increasing due to natural disasters like floods caused by global warming and conflicts resulting from wars. Thus, the driving force for this work was the development of economical and quick material processing technologies to produce customised prosthetic sockets. With this regard, the potential of polyethylene terephthalate (PET) fibre-reinforced composite for fabricating sustainable prosthetic sockets was explored. The choice was defined by its abundant availability in the form of plastic waste from water bottles and food packaging. Thus, rPET-fibres (recycled PET) are readily available in LRNs and are cheaper than synthetic ones.

Many studies were performed to analyse the mechanical behaviour of rPET composites—tensile, flexural, thermal, and fracture [26]. PET-fibre-reinforced composite materials found significant scientific and technological interest related to different applications. Recyclability, combined with the reduced energy use compared to traditional production, resulted in PET fibres becoming a large-volume and value-added product in various sectors, such as construction [27,28,29], textile [30], and medical industries. Furthermore, PET is employed in clinical applications, including cardiovascular grafts, plastic surgery applications, artificial ligaments, and bone augmentation, satisfying the prosthetic bio-compatibility criteria. By varying the crystallisation of PET fibres, a monolithic PETG material was formed that is widely used in AM. The PETG was also used to fabricate the check sockets in some clinical centres [13]. However, like other monolithic polymers, PETG has some potential limitations, such as relatively low limited strength, stiffness, and customisation with a lack of durability.

This paper aims to explore the feasibility of utilising PET-fibre reinforced composite both in woven and knitted fabric to manufacture prosthetic sockets for artificial limb application. The PET-fibre reinforced composites are tested in laminate and socket form to assess their mechanical properties and strength, respectively. Traditional prosthetic socket materials, such as GFRP, HDPE, and PP, used in LRNs were also tested in coupon and socket form for benchmarking purposes.

## 2. Methodology

### 2.1. Materials

**Figure 1:**
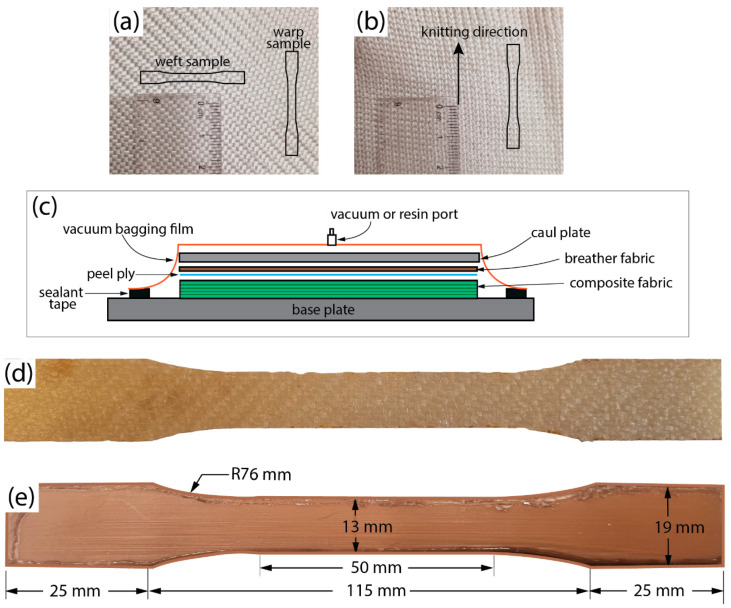
Photographs of woven (**a**) and knitted (**b**) fabric used to fabricate PETW and PETK composites. (**c**) Schematic diagram of VARTM process for manufacturing GFRP, PETW, and PETK composite laminates and Photographs of PETW (**d**) and HDPE (**e**) tensile test specimens used in material characterisation.

The materials used to manufacture PET-fibre-reinforced composite socket for this study was received as woven fabric and yarn spool (supplied by Comfil APS, Gjern, Denmark). The former had a linear density of 440 g/m^2^, while the yarn was 220 tex. The PET-woven (PETW) fabric was cut to the required size to manufacture the laminates or sockets. Using an automated knitting machine, the PET yarn was plain knitted with 12 loops per inch. The composite made using knitted PET fabrics is referred to as PETK in this study. The structure of PETW and PETK fabrics used to manufacture the flat laminates or sockets is shown in Figure 1a,b. For a comparison purpose of materials used in LRNs, sockets were also made of PP, HDPE, and glass-fibre-woven fabric with a linear density of 215 g/m^2^. HDPE was received as pipes from Bhagwan Mahaveer Viklang Sahayata Samiti (BMVSS—an Indian not-for-profit organisation providing low-cost prostheses in Jaipur, India). In all the cases of composite fabrication, IP2 polyester infusion resin (Easy Composites Ltd, Stoke-on-Trent, UK) was used as a matrix material.

### 2.2. Manufacturing and Material Testing of the Laminate

The vacuum-assisted resin transfer moulding (VARTM) process was used to manufacture PETW, PETK, and GFRP composite laminates for material testing. A total of 6 layers of fabrics were stacked with polyester resin impregnated under vacuum. Figure 1c shows the schematic of the VARTM setup used to apply the vacuum pressure on the fabric sheets and resin during manufacturing. The process began by placing the stacked fabric sheets between the two plates, called the *base* and *caul* plates (Figure 1c). The plates were cleaned with acetone to remove dirt. An Ease Release™ 200 Aerosol spray (Bentley Chemicals Ltd., Worcestershire, UK) releasing agent was then sprayed on the plates for easy removal of laminates after the curing process. A breather fabric was used between the *base* and the *caul* plates to remove the excess resin during curing. A vacuum bag was placed over the stacked layers, and their edges were sealed with silicone tape. The negative pressure of 1atm was applied using an external vacuum pump and maintained during the composite consolidation. The polyester resin was mixed with the catalyst according to the manufacturer’s instructions, and the air was removed by degassing the mixed resin. The polyester resin was infused using a resin port. After an hour, the cured composite sample was removed and trimmed with an electrical hacksaw. We followed an identical procedure for curing composite laminates from PETW, PETK, and GF fabric.

Monolithic HDPE flat plates were also made from pipes for direct comparison. The HDPE pipes with a diameter of 200 mm were cut in half and placed in an oven at 200 °C for 40 min. Then, the softened pipes were placed between the rigid platens and pressed in a hydraulic press to yield a uniform thickness of 4 mm. Similarly, a 6 mm thick polypropylene sheet was also preheated in the oven at 160 °C for 40 min, followed by pressing of softened material with the hydraulic press to produce 4 mm flat plate. Preheating of HDPE pipes and polypropylene sheets was performed to ensure that the exact nature of socket materials was used for material characterisation.

An Epilog laser cutter cut dog-bone-shaped specimens from the composite laminates, monolithic HDPE, or PP flat plates. The specimens were subjected to tensile testing following ASTM D638 standard. The specimens were produced with Type V dimensions (Figure 1d,e). Six test samples were prepared for each composite laminate, monolithic HDPE, or PP for tensile testing. In the case of PETK, all the tensile samples were prepared along the knitting loop direction (Figure 1b). However, the PETW laminate tensile strength was tested in both the warp and weft directions of the constituent fabric (Figure 1a). Thus, six samples were made for the PETW laminate (along each warp and weft direction of the constituent fabric), and six samples were made for PETK along the long-knitting direction.

All these samples were tested using the Instron 3369 testing machine at a 1 mm/min rate. The speed of the test was maintained at this level until the failure of the specimen. To measure the strain of the sample accurately, an external strain clip gauge was mounted within the gauge section of the specimen. The force-displacement response of the laminates was recorded during the test. The stress of the material was calculated by determining the force per unit cross-sectional area, while the strain was determined with the strain clip gauge. This stress–strain data were used to compute the modulus ultimate tensile strength of both PETW and PETK materials.

### 2.3. Manufacturing of Test Sockets

**Figure 2:**
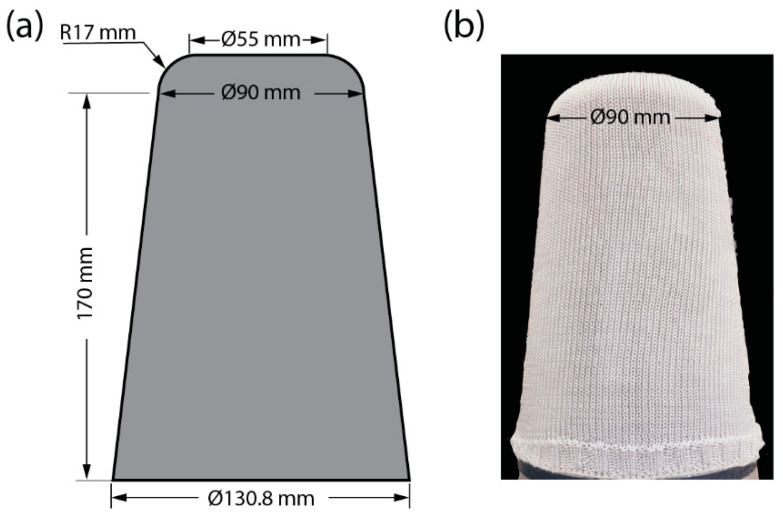
(**a**) Sketch of test socket geometry. (**b**) Photograph of knitted sock used to fabricate PETK sockets.

**Figure 3:**
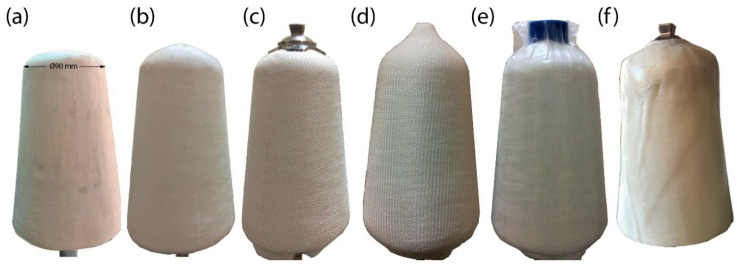
Manufacturing stages for PET-fibre-reinforced composite socket (**a**) preparation of positive cast of test socket using PoP; (**b**) cast with four layers of PET sock; (**c**) pyramid-type adapter placed on the distal end of the cast; (**d**) cast with total eight layers of PET sock; (**e**) resin infiltration during vacuum casting; (**f**) cured PETK socket.

A sketch of the test socket geometry is shown in Figure 2a. The PET yarns were knitted as socks to suit the shape of the test socket geometry (Figure 2b). A total of 8 knitted PET socks were used to manufacture the PETK socket. The VARTM manufacturing sequence followed for the fabrication of the PETK socket is shown in Figure 3. The process began by producing a negative mould with standard dimensions using a Prusa 3D printer. The positive mould was made by pouring the Plaster of Paris into the 3D-printed negative part to fabricate the socket (Figure 3a). The base plate was screwed and cleaned with acetone, and the positive cast was placed on the working table. To avoid chemical reactions between the cast and resin and promote the removal of the final socket from the cast, the cast was wrapped using the cling film, and its surface was treated with the releasing agent before placing PETK socks over the cast. First, four layers of PETK socks were pulled over the cast (Figure 3b), and a pyramid-type connector was placed on the top of the distal end of the cast (Figure 3c). Second, four further layers of PETK socks were pulled over the cast (Figure 3d). Third, the whole cast was sealed with a vacuum bag, and negative pressure of 1 atm was applied using the vacuum pump (Figure 3e). Fourth, the polyester resin was infused into PETK socks using a resin port. Vacuum inside the cast enabled the resin to infiltrate the fabric and improved consolidation in the fabrication process. The negative pressure was maintained for an hour for the resin to thoroughly impregnate the PET socks’ fibres. After an hour, the consolidated PETK socket was removed from the cast (Figure 3f).

An identical VARTM process was followed to manufacture PETW sockets. In this case, the four layers of PET-woven fabric were wrapped around the cast. A pyramid connector was also placed over the cast, and resin was infused under a vacuum. After an hour, the socket was removed from the cast for further testing. Likewise, the VARTM process was repeated to manufacture the polyester resin/glass fibre socket. The socket was fabricated with six layers of cotton socks and one layer of fibreglass woven fabric. Similar to PETK socket fabrication, a positive cast of the socket with standard dimensions was prepared. The cast was covered with cling film, and the releasing agent was sprayed over the cast. Initially, the three layers of cotton socks were stretched over the cast. After this, the pyramid connector was placed at the top of the distal end. The open end of the socks was tied over the base of the pyramid. Following that, the next three layers of cotton socks were pulled over the cast. At last, a fibreglass woven fabric was wrapped around the cast. The whole stack of layers was sealed using the vacuum bag, and negative pressure of 1 atm was applied for one hour. After curing time, the socket was removed from the mould.

**Figure 4:**
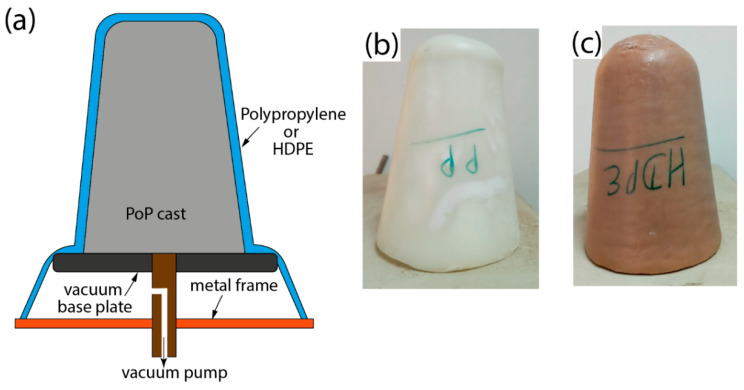
(**a**) Schematic illustration of VAT for manufacturing test sockets using PP and HDPE material and photographs of trimmed PP (**b**) and HDPE (**c**) test sockets.

A vacuum-assisted thermoforming (VAT) process was followed to manufacture the polypropylene socket. It involved draping a preheated polypropylene sheet over the PoP cast mounted on a vacuum base plate. A dry polypropylene sheet was used here instead of moistened PP sheets to manufacture the socket, as the water could react with phosgene in the polycarbonate resin to form CO_2_ gas bubbles. The 6 mm thick PP sheet was secured in a metal annulus and then preheated in the oven until the bubble formed. Then, the metal annulus with the PP bubble was placed over the PoP cast. A sufficient time was allowed until the PP sheet covered the entire length of the cast as well as the vacuum base plate (Figure 4a). It was tied off at the edge of the vacuum base plate before applying negative pressure of 1 atm for an hour. The mould was allowed to cool at room temperature for 1 h before removing the socket from the cast.

The fabrication of the HDPE socket was also similar to that of the PP socket described above. In this case, an HDPE pipe was used instead of a plastic sheet to fabricate the socket. The HDPE pipes with a diameter of 100 mm, length of 150 mm, and thickness of 6 mm were sectioned and preheated in the oven at 200 °C for around 40 min. Then, the preheated pipe was draped over the PoP cast and tied around the edge of the vacuum base plate. A negative pressure of 1 atm was applied for an hour to achieve the cast’s shape. The mould was allowed to cool at room temperature for one hour.

The cured PP and HDPE sockets were trimmed with a power hacksaw to achieve the test geometry (Figure 4b,c). A pyramid-type connector was mechanically fastened at the distal end of the PP and HDPE socket for strength evaluation.

### 2.4. Static Testing of Sockets

**Figure 5:**
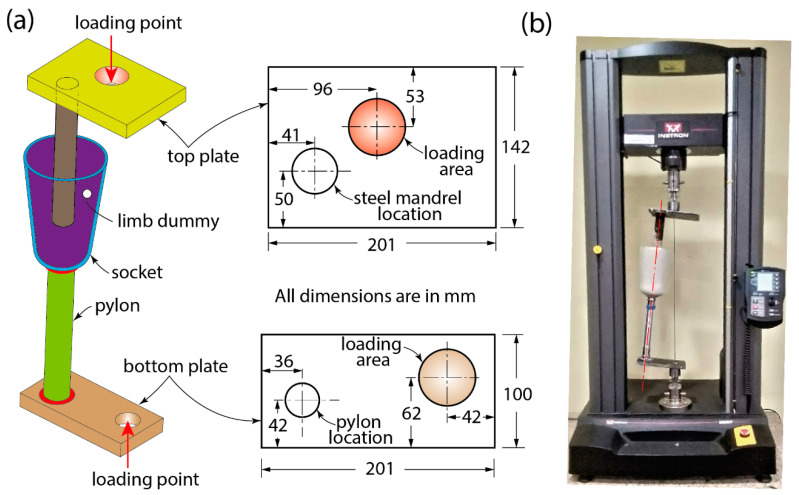
(**a**) Three-dimensional sketch showing various rigid and deformable elements of the test rig to evaluate sockets’ compressive strength (an inset shows the offset distances and other dimensions of the top and bottom plates of the test fixture necessary to simulate forefoot loading during the test). (**b**) Photograph of test socket assembly mounted in a universal testing machine before testing.

A bespoke test fixture was produced for sockets to evaluate their load-bearing capacity, which should exceed 125 kg under static loading (Figure 5). The dimensions of the test fixture were adopted from elsewhere [8,13,31]. Steel plates with 25 mm thickness were machined to produce the top and bottom plates for rigid connections to transfer the load during the compression test. A concave chamfer was machined in both load-application points of the top and bottom plates to facilitate rotation during the loading. To secure the test socket during the test, a steel rod with a 40 mm diameter was welded into the top plate. The open end of the rod was inserted into the socket and aligned so that it was positioned at the mid-section of the socket (Figure 5a) using a bench alignment setup. Then, PoP was poured into the socket and left for 24 h for solidification. The cured PoP cast inside the socket acted as a limb dummy during the compression testing. A pylon adapter was mounted on the bottom plate to secure a 40 mm diameter aluminium pylon. The pylon was mechanically fastened to the pyramid connector attached to the distal end of the socket.

The final assembly of the socket-testing fixture before testing is shown in Figure 5b. Apparently, the load line is inclined to the vertical axis of the machine. The offset distance of the load-application points of the top and bottom plates enabled the inclination load line; thus, it generated larger moments at the distal end of the sockets, simulating forefoot loading, according to the ISO 10328 standard [32]. An identical procedure was followed for each test to create the limb dummy and new socket connectors and pylons to directly compare each socket’s performance.

In this part of the study, 15 sockets (3 for each socket material) were tested using the Instron 5967 Universal testing machine to check their capability to the load corresponding to 125 kg (twice the average body mass of an amputee residing in LRNs). This body mass limit was imposed due to the load rating of socket connectors and the aluminium pylon used in this study. The load was applied at a rate of 10 N/s during the test until it passed 1250 N, followed by the unloading.

## 3. Results

### 3.1. Composite Architecture

**Figure 6:**
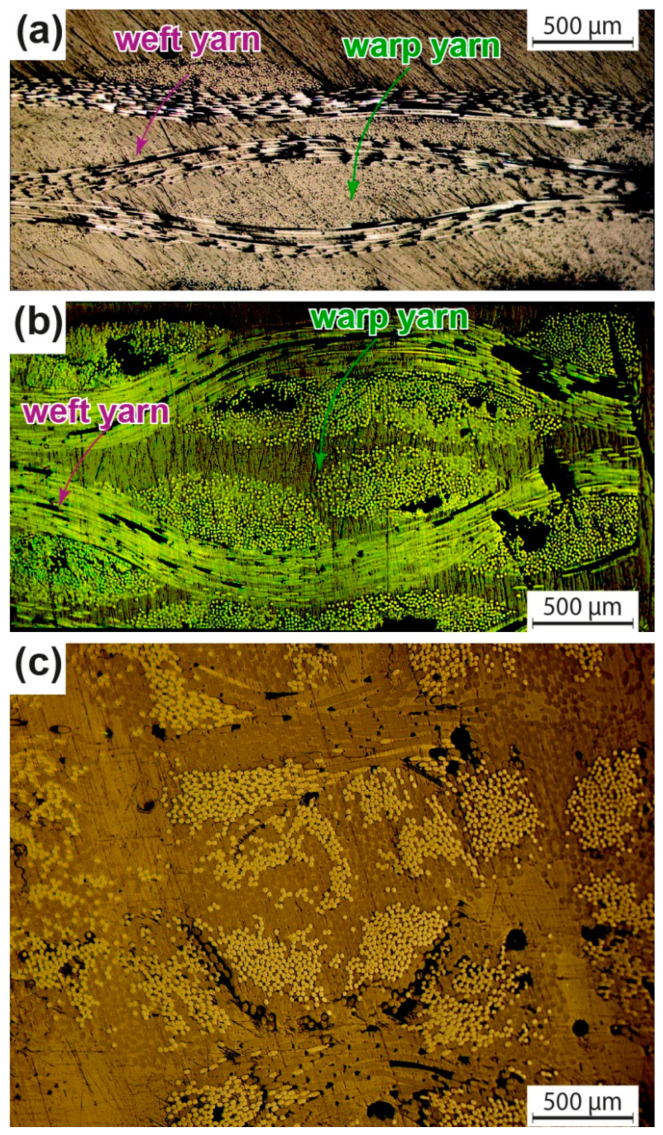
Cross-section microscopy images for the GFRP (**a**), PETW (**b**), and PETK (**c**) composites.

We examined the composite specimens after curing by sectioning and polishing them to obtain their architecture. To create the full-field view in Figure 6, we combined 20 cross-sectional images using the Nikon Eclipse LV150N microscope equipped with an automatic focus. Figure 6a,b show that the warp and weft direction fibres are visible for the GFRP and PETW composite, demonstrating that the polyester matrix serves as a binding agent for the reinforcing fibres without any other interaction. Moreover, Figure 6 indicates that the reinforcement fibres were well-encapsulated within the matrix, and the adjacent layers adhered properly during the VARTM process. Due to the vacuum used during consolidation, the composite had fewer voids, making the VARTM process suitable for fabricating PET-fiber-reinforced composites. Figure 6c shows that the fibre reinforcements in PETK composite were evenly distributed compared to GFRP and PETW composite.

### 3.2. Tensile Response of Socket Materials

**Figure 7:**
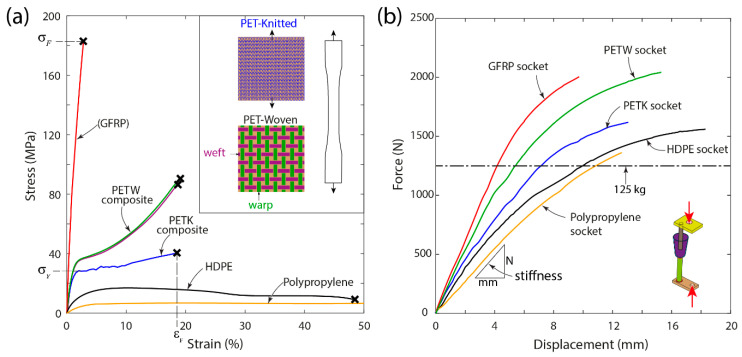
Measured tensile response of socket materials (**a**) and structural response of test sockets (**b**) under compressive loading.

The measured tensile stress–strain response of socket materials is presented in Figure 7a. Two distinct types of response were observed, an elastic-brittle response of GFRP composite and an elastic-plastic response for PET composites as well as the monolithic PP and HDPE materials. The GFRP composite had the highest level of strength and stiffness among all the materials tested in this study. The PETW laminates demonstrated identical responses in both the warp and weft directions of the constituent fabric (Figure 7a). The yield strength of PETW and PETK laminates was identified; however, their magnitude depended upon the fabric architecture. This was also true for the strength and strain at failure of PETW and PETK laminates. This demonstrates that the structure of the constituent fabric (woven and knitted) plays an essential role in defining the mechanical properties of the laminate.

The summary of measured mechanical properties, such as Young’s modulus, yield strength, strength, and strain at failure for all socket materials tested in this study, is presented in Table 1. The GFRP composite laminate had the highest stiffness of 12.1 GPa and failure strength of 178 MPa. The PETW, PETK, PP, and HDPE materials had similar stiffness levels ranging from 0.3 to 3.6 GPa, which were significantly lower than those of the GFRP composite. The strength of the PETW laminate is approximately two times higher than that of the PETK laminate. The yield stress and strength of monolithic HDPE and PP materials were much lower than those of PET fibre-reinforced composites, with the latter being the weakest among other socket materials in this study. These results show that PET fibre-reinforced composite materials are stronger and tougher than the widely used monolithic thermoplastic socket materials.

**Figure 8:**
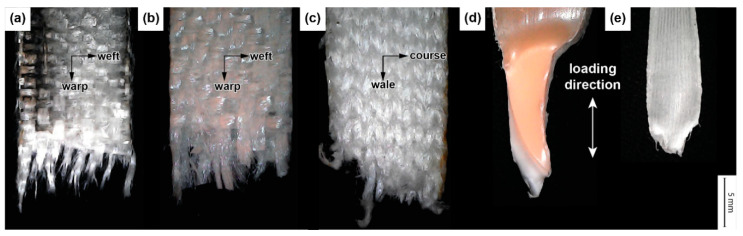
Photographs of GFRP (**a**), PETW (**b**), PETK (**c**), HDPE (**d**), and PP (**e**) samples, which failed in the tensile test. An inset featuring a microscopy image demonstrates the material failure.

**Figure 9:**
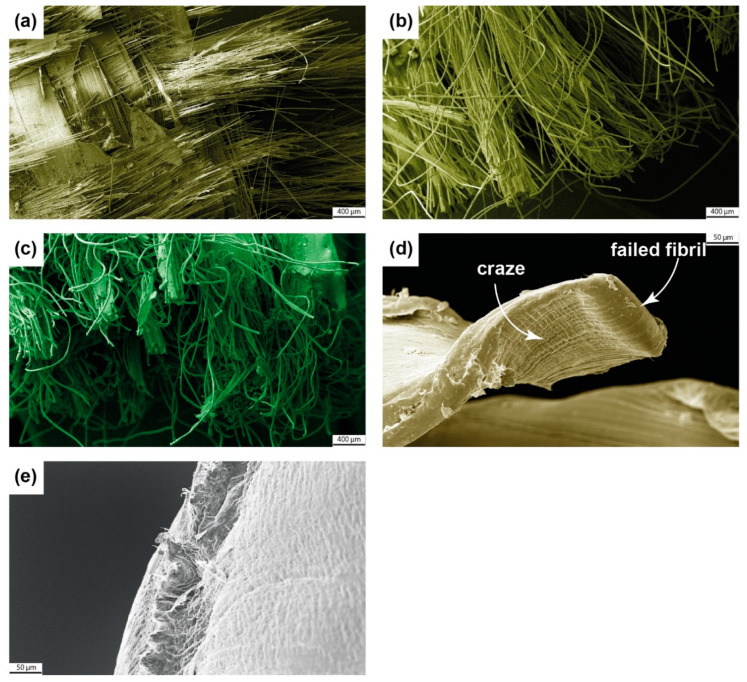
Scanning Electron Microscopy images of GFRP (**a**), PETW (**b**), PETK (**c**), HDPE (**d**), and PP (**e**) samples featuring the material failure under tensile loading.

Figure 8 displays the failed end of the tensile test specimens. We observed fibre fracture along the loading direction across all composite specimens. In the woven composite (GFRP and PETW), the reinforcing fibres in the warp direction suffered a fracture, while the fibres in the weft direction were displaced without any fibre failure. In the knitted composite (PETK), the fibres in the wale direction realigned to the loading direction and eventually failed upon reaching the failure stress. The scanning electron microscopy images of the failed end of the composite are shown in Figure 9a–c. It is worth noting that all composite specimens primarily failed due to fibre fracture.

In the monolithic HDPE and PP samples, a classic cup–cone type ductile failure [33,34] was observed (as shown in Figure 8d,e). This occurred when the material was stretched within its gauge section during the tensile loading process. As a result, cracks initiated and grew, which led to the formation of crazes on the surface of the specimens. These crazes are small, localised, plastically deformed zones with dense fibrils separated by microvoids. Upon closer inspection of the HDPE sample in Figure 9d, it can be seen that there were crazing and recoiled failed fibrils after the tensile failure. On the other hand, the PP sample failed surface had a peak and valley, indicating that the final failure was due to microvoid coalescence [33,34].

### 3.3. Socket Weight and Shape

Socket weight is a crucial parameter for the selection of the right socket. Although there is limited information related to a link between the socket weight and the patient’s gait or metabolic energy consumed while walking, a lighter socket is always preferred by amputees. The weight of the prosthetic sockets used in this study is presented in Table 2. The ranking of test socket weight is as follows: PP < PETK < HDPE < PETW < GFRP. Notably, the weight of the PETW and PETK sockets was similar to that of commonly used HDPE ones. The GFRP sockets had a higher weight than the others due to the six layers of cotton socks used during the lamination process. Thanks to its higher stiffness, it is possible to reduce the layers of the GF woven fabrics; therefore, the total cost and weight of the GFRP socket can be decreased.

In terms of the shape of the socket, traditionally manufactured sockets, such as HDPE and PP ones, are made by technicians using labour-intensive methods. As a result, some slight variations in socket shapes are possible. However, the sockets based on PET yarns were made with socks made with the automatic knitting machine that reduces the variation between sockets and provides repeatability. As a result, a better shape can be achieved consistently via this process. Another potential benefit of using the PETK based on automatic knitting is the possibility of personalisation of the socket manufacturing process to reduce the manufacturing time. The PETK socks with the required dimensions can be knitted based on the patients’ digital data for manufacturing patient-specific sockets.

### 3.4. Static Structural Test of Sockets

The measured force-deflection response of the test sockets tested under compressive loading is presented in Figure 7b, demonstrating the average responses obtained for three test sockets. All the sockets exhibited a monotonic increase in the load with growing displacement during the initial stage of the test. Beyond 1000 N, this monotonic increase was followed by a softening response. At this stage, the aluminium pylon of the test rig began to deform, causing this softening response. All the test sockets could withstand the target load-bearing capacity of 125 kg (marked in Figure 7b) without failure. The total displacement measured at 125 kg load is presented in Table 2. The monolithic PP and HDPE sockets exhibited a displacement excess of 9 mm, whereas the composite sockets (GFRP, PETW and PETK) showed better resilience to the deformation than their monolithic counterparts.

To quantify the prosthetic socket stiffness (N/mm), the slope of the initial linear portion of the force-displacement response (see Figure 7b) was calculated for each socket by curve fitting with the second-order polynomial (Table 2). The monolithic PP, HPDE, and PETK socket showed the lowest stiffness in a 135–159 N/mm range. The highest stiffness of 319 N/mm was obtained from GF composite socket, whereas the PETW socket exhibited an intermediate stiffness of 233 N/mm. Since these magnitudes were obtained for sockets of various weights and thicknesses, a specific parameter defined as the ratio of stiffness to mass was also calculated (Table 2). The GFRP, HDPE, and PP sockets had similar levels of specific stiffness in a range from 0.49 to 0.53 $\frac{N/mm}{g}$, which was significantly lower than that of the PET-fibre-reinforced ones.

## 4. Discussion

This exploratory study’s main goal was to investigate the possibility of using PET-fibre-reinforced composites for manufacturing prosthetic sockets and the socket performance compared with their counterparts made from socket materials commonly available in LRNs. This was achieved by characterising the mechanical properties of the socket materials and structural testing of the produced sockets. The experimental campaign revealed that the PET-fibre-reinforced composite could replace the currently used socket materials in LRNs.

The tensile tests on socket materials demonstrated that the PETW and PETK laminates had superior material properties compared to those of the monolithic PP and HDPE. The architecture of PET fabric (woven or knitted) played a crucial role in governing the mechanical properties of its composites. It found that an important parameter influencing the material property was the knitting structure of the yarns. Therefore, carefully selecting the knitting pattern is crucial to obtain the required mechanical performance of the material and will be explored in future studies.

The static structural testing of test sockets revealed that they all achieved the target load-bearing capacity of amputees’ double average body mass residing in LRNs. The GFRP, PETW, and PETK sockets had better resistance to deformation compared to their monolithic counterparts made from PP and HDPE. Furthermore, the PETW and PETK sockets had a higher level of specific stiffness than their counterparts from GFRP, HDPE, and PP. Therefore, PET-fibre-reinforced composites can replace monolithic socket materials to improve the long-term durability of prostheses.

The material cost of PEW and PETK fabrics are similar to GFRP, HDPE, and PP materials commonly used in LRNs (Table 2). In comparison, PETW and PETK fabrics cost significantly lower than 2/2 twill woven carbon fibre fabric with a linear density of 240 g/m^2^, costing between $137 to $174 on average. As a result, utilising PET-fibre-reinforced composites is a cost-effective option for creating prostheses for LRNs.

This study has limitations. The feasibility of PET-fibre-reinforced composite as an alternative composite material to replace the prohibitively expensive composites made with carbon, glass, and Kevlar fabrics, as well as less-durable monolithic thermoplastics, such as PP and HDPE, used in LRNs was explored. Our objective was to develop affordable and durable prosthetic sockets that aid the essential mobility of amputees still without their first prosthesis. For this reason, the socket’s load-bearing capacity of 125 kg was considered. Unfortunately, it was not feasible to evaluate the ultimate load-carrying capacity of sockets due to the bending of the aluminium pylon.

Further work with a better pylon is needed to test the socket’s ultimate strength according to ISO 10328 standards. The exact VARTM process was used to manufacture the PET-fibre-reinforced sockets for simplicity and consistency in manufacturing laminated sockets as practised in LRNs. Future studies are, therefore, required to adopt the VAT process to heat-cure the PETW and PETK laminates with different fabric architectures. This can significantly improve the specific mechanical properties of sockets made of these laminates. In addition, it is necessary to conduct cyclic tests on sockets manufactured from PET fibre-reinforced composite to assess their durability. A comparative study measuring the strength of these sockets against commercially available ones would also be beneficial for prosthetic applications. Despite the study’s shortcomings, the results already showed that PET-fibre-reinforced composites are better, low-cost, sustainable materials for manufacturing prostheses.

## 5. Conclusions

Although a prosthetic socket’s fit and function are crucial for a patient’s comfort, its strength should not be underestimated. Moreover, the ease of customisation and fabrication of the prosthetic socket is equally crucial for prosthetists. To address all these aspects, we looked into the possibility of substituting the traditional composite materials utilised in the production of prosthetic-limb sockets with composites reinforced by PET fibres. Direct comparison was also performed with traditional glass-fibre-reinforced composites and monolithic thermoplastics used in prosthetic socket fabrication, such as PP and HDPE.

Our tensile-strength tests indicated that PET-fibre-reinforced composite made from woven and knitted fabrics could produce a strong socket material. The static tests performed on the PETW and PETK sockets showed higher stiffness and deformation resistance than those made from monolithic PP and HDPE. Therefore, PET-fibre-reinforced composites are an ideal choice to replace those materials to develop durable prosthetics for amputees in LRNs. According to the study, prosthetic sockets reinforced with PET fibres can withstand the required static strength. To evaluate their effectiveness, field trials with amputees are recommended.

Knitting to produce the desired near-net shape of the socket demonstrated outstanding potential for producing customised prosthetic sockets. This technique provides ease of customisation to manufacture the socket using PET-yarn and is a step towards automation in socket fabrication.

## Figures and Tables

**Table 1:** Measured properties of socket materials used in this study.

Parameters	PETW	PETK	GFRP	PP	HDPE
**Young’s modulus** $\left(E_{1},\mathrm{GPa}\right)$	3.60 ± 0.17	3.16 ± 0.14	12.1 ± 1.49	0.3 ± 0.04	0.86 ± 0.07
**Yield strength** $\left(\sigma_{y},MPa\right)$	34.27 ± 0.52	20.05 ± 0.35	-	6.24 ± 0.49	16.01 ± 0.17
**Failure stress** $\left(\sigma_{F},MPa\right)$	90 ± 1.88	41.18 ± 0.92	178.77 ± 2.81	7.4 ± 0.48	17.17 ± 0.22
**Strain to failure** $\left(\varepsilon_{F},\%\right)$	18.85 ± 0.28	18.5 ± 0.17	2.75 ± 0.05	>50%	49 ± 0.96

**Table 2:** The measured mechanical and physical properties of the sockets tested in this study.

Socket	Wall Thickness at Distal End (mm)	Stiffness (N/mm)	Displacement at 1250 N (mm)	Socket Weight (g)	**Specific Stiffness** $\left(\frac{N/mm}{g}\right)$	Cost $/kg
GFRP	4.41 ± 0.2	319 ± 3	4.77 ± 0.06	595	0.5361	15–54
PETW	5.80 ± 0.1	233 ± 2	5.37 ± 0.23	297	0.7845	13
PETK	5.50 ± 0.1	159 ± 5	7.16 ± 0.12	245	0.6490	13
HDPE	5.10 ± 0.4	140 ± 2	9.54 ± 0.80	283	0.4947	4–15
PP	6.60 ± 0.3	135 ± 1	10.91 ± 0.10	261	0.5172	20–86

## Data Availability

The data presented in this study are available on request from the corresponding author.
